# Effects of Echinacoside on Ehrlich Carcinoma in Rats by Targeting Proliferation, Hypoxia and Inflammation

**DOI:** 10.7759/cureus.46800

**Published:** 2023-10-10

**Authors:** Afnan Alshehri, Aeshah Albuhayri, May Alanazi, Manal A Althubaiti, Raghad F Aljehani, Fai I Alsharif, Taghreed M Alatawi, Shouq S Albalawi, Ahmed E Khodir, Mohammed M Al-Gayyar

**Affiliations:** 1 PharmD Program, Faculty of Pharmacy, University of Tabuk, Tabuk, SAU; 2 PharmD Program, University of Tabuk, Tabuk, SAU; 3 Pharmacology and Toxicology, Horus University, New Damietta, EGY; 4 Pharmaceutical Chemistry, Faculty of Pharmacy, University of Tabuk, Tabuk, SAU; 5 Biochemistry, Faculty of Pharmacy, Mansoura University, Mansoura, EGY

**Keywords:** tumor necrosis factor (tnf)-α, phosphoinositide-3-kinases (pi3k), nuclear factor (nf)κb, mammalian target of rapamycin (mtor), hypoxia-inducible factor (hif)-1α, ehrlich solid carcinoma (esc), cyclin-dependent kinase 2 (cdk2), cyclin d1

## Abstract

Background and objectives

Ehrlich solid carcinoma (ESC) is a type of tumor originating from a spontaneous mammary adenocarcinoma in mice. It is highly aggressive and fast-growing and can create a solid undifferentiated mass when inserted under the skin. This makes it an ideal model for assessing cancer biology and tumor immunology. Echinacoside is a natural phenylethanoid glycoside with anti-inflammatory, anti-endoplasmic reticulum stress, anti-oxidative stress, and other beneficial properties. This study explored the potential anti-cancer benefits of echinacoside in rats with ESC. The study also analyzed its effects on tumor cell proliferation, differentiation, motility, and inflammation.

Methods

The study involved injecting rats with tumors in their left hind limb using an intramuscular injection of 2×10^6^ cells. After 14 days, some rats were given a daily intraperitoneal dose of 30 mg/kg echinacoside for three weeks. Muscle samples were then analyzed under an electron microscope. In addition, gene expression and protein levels of various factors such as phosphoinositide 3-kinases (PI3K), mammalian target of rapamycin (mTOR), hypoxia-inducible factor (HIF)-1α, cyclin D1, cyclin-dependent kinase 2 (CDK2), tumor necrosis factor (TNF)-α, and nuclear factor (NF)κB were evaluated in another part of the muscle samples.

Results

After being treated with echinacoside, the ESC rats experienced a significant increase in their mean survival time from 27 days to 48 days. This treatment also resulted in a decrease in the volume and weight of the tumor. Upon examining the tumor tissue under an electron microscope, signs of damage such as pleomorphic cells, necrosis, nuclear fragmentation, membrane damage with cytoplasmic content spilling, and loss of cellular junction were observed. However, the treatment with echinacoside was effective in improving these effects. Furthermore, the expression of PI3K, mTOR, HIF-1α, cyclin D1, CDK2, TNF-α, and NFκB was significantly reduced due to the echinacoside treatment.

Conclusions

Our research found that echinacoside has antitumor properties that resulted in a substantial decrease in tumor size and weight, leading to an increase in the average survival time of rats and an improvement in muscle structure. Additionally, echinacoside was shown to ameliorate hypoxia by suppressing HIF-1α, reduce inflammation by decreasing NFκB and TNF-α, decrease proliferation by reducing PI3K, and block cyclin D1 and CDK2 to inhibit differentiation.

## Introduction

Coping with cancer is a complex issue that poses several challenges due to the nature of the disease and the side effects of chemotherapy. These side effects can include hair loss, fatigue, and nausea, among other things, making cancer the second most significant cause of death after heart disease. It is projected that about 1.9 million new cancer cases and 609,360 deaths from cancer occur in the US in 2022, resulting in an average of 1,670 deaths every day [1]. To address the shortcomings of traditional cancer treatments, much emphasis has been placed on developing new treatments. One method of studying tumor biology and evaluating new anticancer drugs is using the Ehrlich solid carcinoma (ESC) model. This type of tumor is an undifferentiated solid originating from a spontaneous murine mammary adenocarcinoma and is an aggressive and fast-growing carcinoma that can form a solid mass when inoculated subcutaneously [2].

The Phosphoinositide 3-kinase (PI3K) is crucial in regulating cell growth and metabolism. The significance of PI3K in cancer development was first suggested in 1985 and has since been extensively studied. PI3K belongs to a family of lipid kinases that play a unique role in signal transduction. It has a significant impact on various types of tumors, including leukemia, lung cancer, and breast cancer [3].

Recently, there has been an increasing interest in creating new medicines derived from plants as an alternative to traditional cancer treatments. One of the main reasons for this interest is their effectiveness, combined with their lower toxicity compared to conventional chemotherapy. Echinacoside is an active compound that is derived from both *Cistanches Herba* and Echinacea. It has been found to produce various biological effects, including neuroprotection, immune regulation, and anti-tumor properties [4]. In addition, studies have shown that echinacoside can inhibit the proliferation, invasion, and migration of liver cancer cells (HepG2) in vitro [5]. However, there has been no previous research on the ability of echinacoside to produce therapeutic effects against ESC. To address this gap, we conducted a study to explore the potential anti-cancer benefits of echinacoside in rats with ESC. Our study also analyzed the compound's effects on ameliorating hypoxia by suppressing hypoxia-inducible factor (HIF)-1α, reducing inflammation by decreasing nuclear factor κB (NFκB) and tumor necrosis factor-α (TNF-α), decreasing proliferation by reducing PI3K, and blocking cyclin D1 and cyclin-dependent kinase 2 (CDK2) to inhibit differentiation.

## Materials and methods

Animals and treatment outlines

Our study was conducted on 36 Sprague-Dawley rats weighing 180-200 g. These rats were kept in standard temperature conditions and followed a 12-hour light/12-hour dark cycle. The research ethics committee at the Faculty of Pharmacy, Horus University approved the work protocol under number (P2023-003). The rats were divided into three groups, with 12 rats in each group. The control group (no ESC nor echinacoside) did not receive any treatment during the experiment. The ESC group (ESC with no echinacoside) was injected with 200 µL of 2x10^6^ tumor cell suspension in phosphate buffer saline into the left hind limb using intramuscular injection [6-8]. In the ESC+echinacoside group, the first tumor was injected in rats and left to grow for 14 days, the time taken for the tumor to appear. Then, on day 15, rats were given 30 mg/kg of echinacoside (Sigma Aldrich Chemicals Co., St Louise, MO, USA) in water by intraperitoneal injection. Rats were treated with echinacoside for three weeks.

Echinacoside was not used previously to attenuate ESC. Therefore, preliminary studies tested four different concentrations of echinacoside 20, 30, 40, and 50 mg/kg. The dose of 30 mg/kg was selected as it was found to be the lowest concentration that produced therapeutic effects.

Sample collection

Out of the 12 rats in each group, nine rats were sacrificed after five weeks of the experiment. The three rats left were kept until they passed away to calculate the survival time. The entire tumor region on the left hind leg's thigh was removed, measured, and weighed. A portion of the muscle tissue was treated with 10% buffered formalin and analyzed through morphological and immunohistochemical investigations. Another part of the tissue was homogenized in a sodium potassium phosphate buffer (0.01 M, pH 7.4) containing 1.15% KCl, at a 10-fold volume and kept in cold temperature. The resulting supernatant was stored at -80°C.

Specimen preparation for transmission electron microscopy and immunohistochemistry

Samples larger than 1 mm^3^ were isolated and treated with glutaraldehyde at a temperature of 4°C for 4 hours. Following this, the samples were dehydrated using a graded series of ethanol and propylene oxide before being embedded in epoxy resin. To observe ultrathin sections, they were visualized at 160 kV using a JEOL JEM-2100 at the Electron Microscope Unit, Mansoura University, Egypt. Skin tissue was used to create five-micrometer thick paraffin blocks, which were then immunostained with monoclonal anti-PI3K (Sigma Aldrich Chemicals Co.) at 4°C [9-11].

Enzyme-linked immunosorbent assay (ELISA)

The levels of PI3K, mTOR, HIF-1α, cyclin D1, CDK2, and TNF-α were assessed using commercially available ELISA kits from MyBioSource, Inc. in San Diego, CA, USA. The manufacturer's instructions were followed for the assessment.

Quantitative real-time polymerase chain reaction (RT-PCR)

To examine the mRNA expression of various genes in rat skin lysates, we utilized previously described methods [12-15]. We used GAPDH as a housekeeping gene and internal reference. The specific PCR primers used for each gene are listed in Table 1.

**Table 1:** The primers set used to detect gene expression in rats

Name	Sequence		Reference sequence
GAPDH	Forward	5`-CCATCAACGACCCCTTCATT-3`	NM_017008.4
	Reverse	5`-CACGACATACTCAGCACCAGC-3`	
HIF-1α	Forward	5`-GCAACTAGGAACCCGAACCA-3`	NM_024359.2
	Reverse	5`-TCGACGTTCGGAACTCATCC-3`	
PI3K	Forward	5′-TTACGGCGGCATGGGAATCT-3′	XM_017595947.2
	Reverse	5′-CCAGCTTTCCCTGAGTGCCT-3	
NFκB	Forward	5`-TCTGTTTCCCCTCATCTTTCC-3`	AF079314.2
	Reverse	5`-GCGTCTTAGTGGTATCTGTGCTT-3`	
TNF-α	Forward	5`-AAATGGGCTCCCTCTCATCAGTTC-3`	X66539
	Reverse	5`-TCTGCTTGGTGGTTTGCTACGAC-3`	
mTOR	Forward	5′-CTGCACTTGTTGTTGCCTCC-3′	NM_019906.2
	Reverse	5′-ATCTCCCTGGCTGCTCCTTA-3′	
Cyclin D1	Forward	5′-TCGACGGCCATTACCAATCG-3′	X75207.1
	Reverse	5′-CGCAGACCTCTAGCATCCAG-3′	
CDK2	Forward	5`- GAGCTCTGCTTGCGTTCCATC-3`	NM_199501.2
	Reverse	5`- GGGTCACCATTTCGGCAAAG-3`	

Statistical analysis

In this study, quantitative variables were presented as mean ± standard error. To compare the significance between groups, we used a one-way analysis of variance (ANOVA). If significance was found, we used the post hoc Bonferroni correction test. All statistical analyses were performed using SPSS version 20 (IBM Corp., Armonk, NY, USA). We defined statistical significance as P < 0.05.

## Results

Antitumor activity of echinacoside

**Figure 1:**
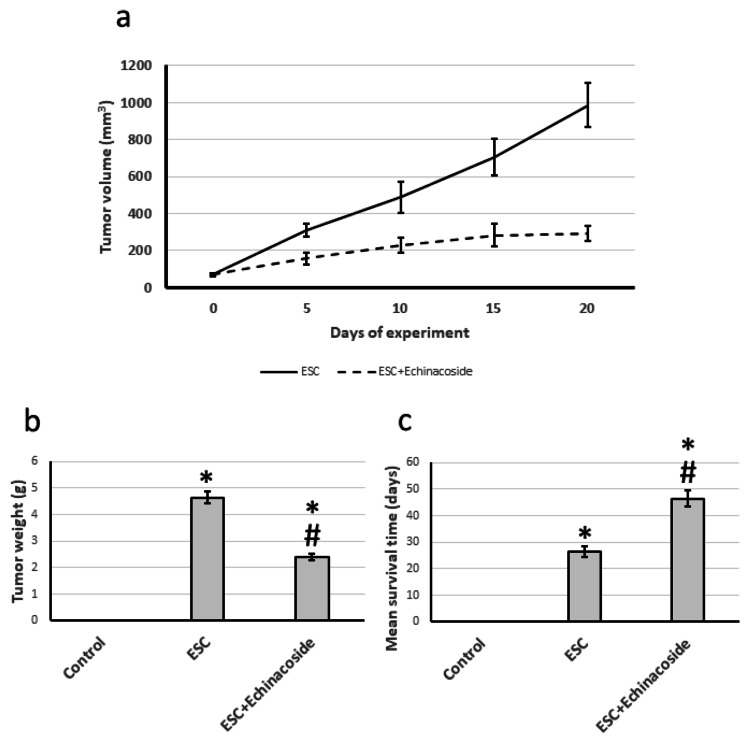
Investigating the impact of ESC and 30 mg/kg echinacoside on tumor volume (a), tumor weight (b), and rat survival time (c). * There was a significant difference between the examined group and the control group at p<0.05. # There was a significant difference between the examined group and the ESC group at p<0.05. ESC: Ehrlich solid carcinoma.

Throughout the course of the experiment, we observed a marked rise in tumor volume and cancer weight on the day of sacrifice. However, we found that treatment with echinacoside in the ESC group led to a significant reduction in both tumor volume and weight when compared to the control group. Figure 1 demonstrates that the use of echinacoside also resulted in a noteworthy increase in the average survival period of ESC rats, from 27 to 48 days.

Effect of echinacoside on the morphology of muscle tissues

**Figure 2:**
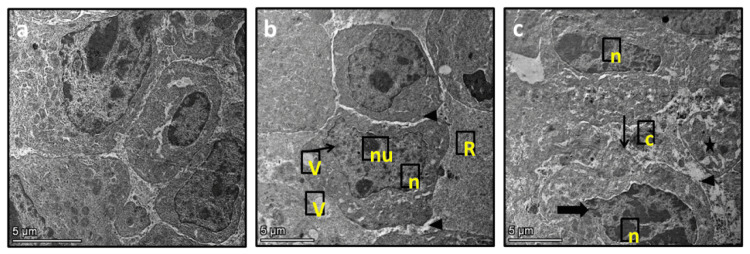
The muscle sections were examined under an electron microscope in three groups: the control group (a), the ESC (b), and the ESC group treated with 30 mg/kg echinacoside (c). The images revealed changes in the cytoplasm, with multivesiculated bodies (V) attached to the plasma membrane. The nucleus (n) showed irregularity with less segmentation and two nuclei (nu) with scattered interchromatin granules (thin arrow). ESC: Ehrlich solid carcinoma.

Under the influence of ESC, micro images depict cytoplasmic changes characterized by the emergence of multivesiculated bodies (V) attached to the plasma membrane. The nucleus (n) exhibits irregularity with less segmentation and two nuclei (nu) display scattered interchromatin granules (thin arrow). The treatment of ESC rats with 30 mg/kg echinacoside ameliorated all these effects (Figure 2).

Effect of echinacoside on PI3K expression

**Figure 3:**
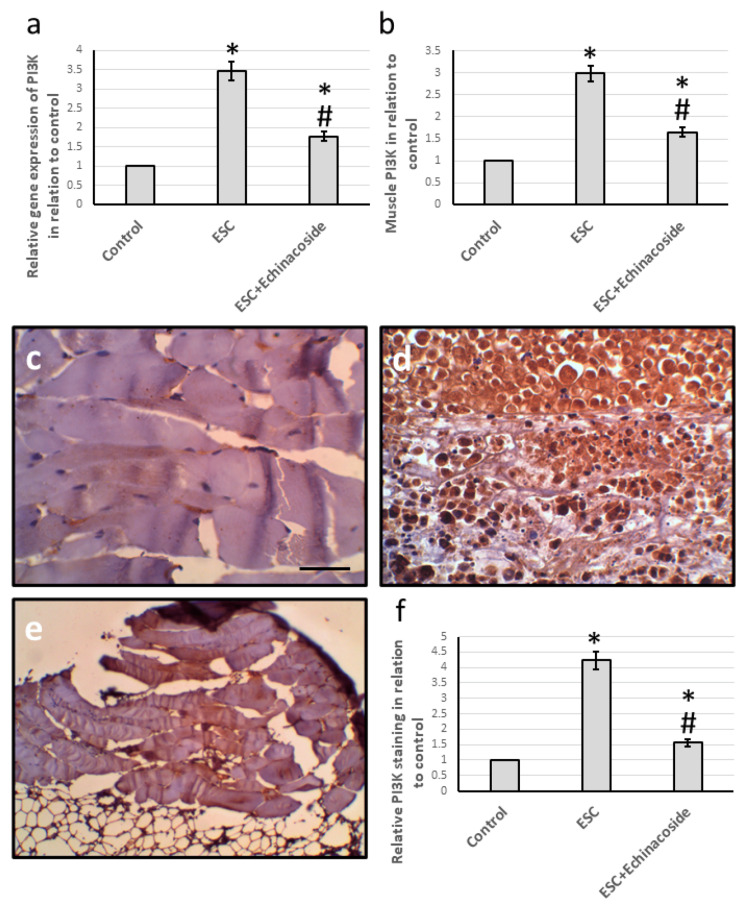
The examination of the impact of ESC and 30 mg/kg echinacoside on the expression of the PI3K gene (a) and protein level (b). Additionally, muscle sections were analyzed and stained with anti-PI3K antibodies for the control group (c), ESC group (d), and ESC group treated with echinacoside (e). The positive staining score was determined through immunohistochemistry in ten different fields (f). * There was a significant difference between the examined group and the control group at p<0.05. # There was a significant difference between the examined group and the ESC group at p<0.05. ESC: Ehrlich solid carcinoma; PI3K: Phosphoinositide 3-kinases Scale bar is 50 µm.

The lipid kinase family known as phosphoinositide 3-kinases (PI3Ks) is crucial in regulating various cellular processes, such as cell survival, proliferation, and differentiation. In comparison to the control group, ESC was found to increase both PI3K gene expression and muscle protein levels. The analysis of muscle sections stained with anti-PI3K antibodies revealed an increase in ESC, while echinacoside treatment resulted in a significant decrease in gene expression, protein levels, and immunostaining (Figure 3).

Effect of echinacoside on the expression of mTOR and HIf-1

**Figure 4:**
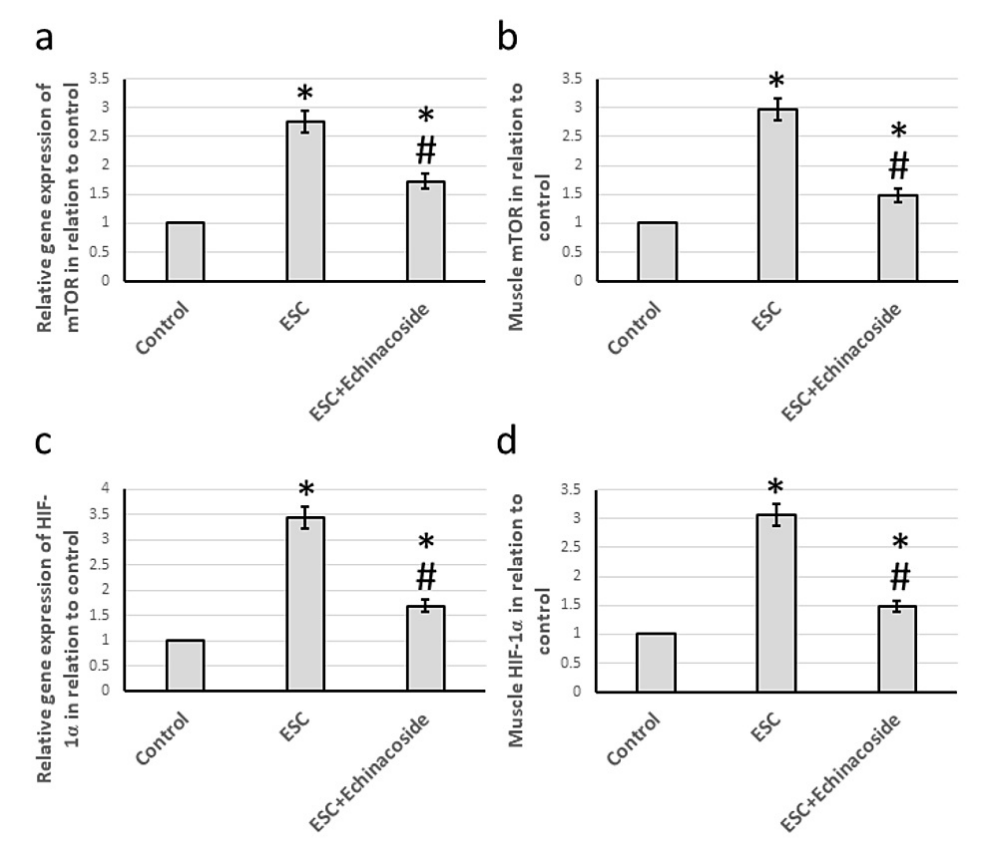
Effect of Ehrlich solid carcinoma (ESC) and 30 mg/kg echinacoside on gene expression of mTOR (a) and HIF-1α (c) as well as the muscle level of mTOR (b) and HIF-1α (d). * There was a significant difference between the examined group and the control group at p<0.05. # There was a significant difference between the examined group and the ESC group at p<0.05. ESC: Ehrlich solid carcinoma; HIF-1α: Hypoxia-inducible factor-1α; mTOR: mammalian target of rapamycin.

In comparison to the control rats, the administration of ESC led to a significant increase in the gene expression of mTOR and HIF-1α by 2.76- and 3.44-fold, respectively. Additionally, ESC resulted in a remarkable elevation in the muscle protein levels of both mTOR and HIF-1α by 2.97- and 3.06-fold, respectively. However, when echinacoside treatment was given, it reduced the gene expression and muscle protein levels of both mTOR and HIF-1α in ESC rats, although still higher than the control group as shown in Figure 4.

Effect of echinacoside on the expression of cyclin D1 and CDK2

**Figure 5:**
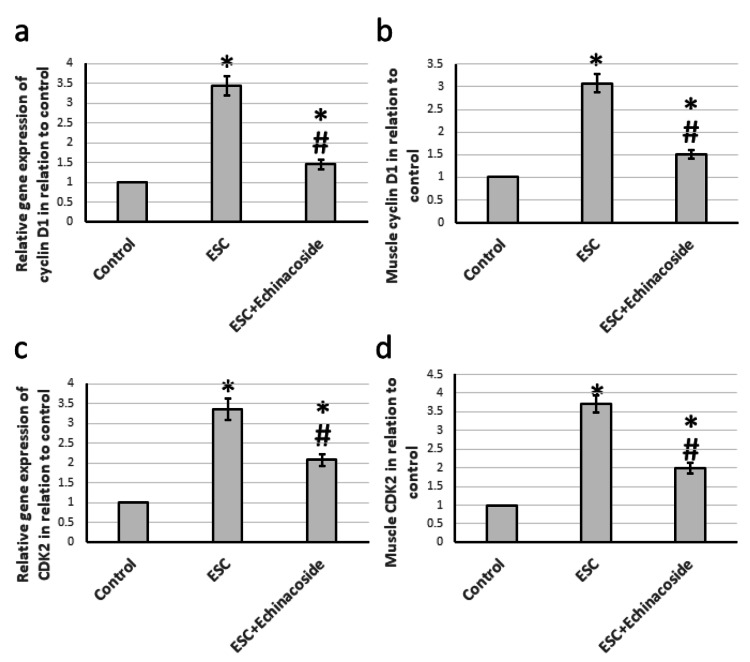
Effect of ESC and 30 mg/kg echinacoside on the expression of Cyclin D1 (a) and CDK2 (c), as well as the muscle levels of Cyclin D1 (b) and CDK2 (d). * There was a significant difference between the examined group and the control group at p<0.05. # There was a significant difference between the examined group and the ESC group at p<0.05. ESC: Ehrlich solid carcinoma; CDK2: Cyclin-dependent kinase 2.

When compared to the control group of rats, those that were given ESC experienced a significant increase in the expression of cyclin D1 and CDK2 genes by 3.43- and 3.36-fold, respectively. Muscle protein levels of both cyclin D1 and CKD2 also saw notable elevation by 3.08- and 3.72-fold, respectively. However, the introduction of echinacoside treatment resulted in a decrease in gene expression and muscle protein levels of mTOR and HIF-1α in ESC rats, although still higher than the control group, as illustrated in Figure 5.

Effect of echinacoside on the inflammatory pathway

**Figure 6:**
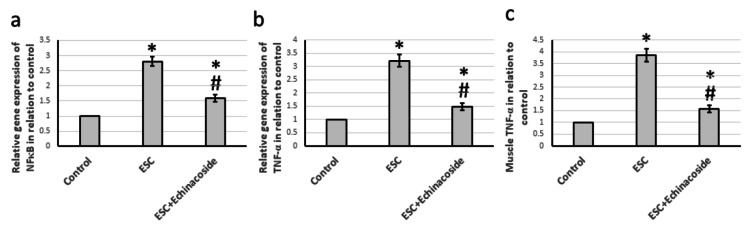
Effects of ESC and 30 mg/kg echinacoside on the expression of NFκB (a) and TNF-α (b), as well as the muscle TNF-α level (c). * There was a significant difference between the examined group and the control group at p<0.05. # There was a significant difference between the examined group and the ESC group at p<0.05. ESC: Ehrlich solid carcinoma; NFκB: Nuclear factor κB; TNF-α: Tumor necrosis factor-α

According to the findings, ESC led to a noteworthy rise in the gene expression of NFκB and TNF-α by 2.81- and 3.22-fold, correspondingly, compared to the control group. Additionally, ESC resulted in a 3.86-fold increase in TNF-α muscle protein levels, as opposed to the control group. On the other hand, echinacoside therapy showed a considerable decrease in gene expression and muscle protein levels of both NFκB and TNF-α, compared to ESC, but still higher than the control group (refer to Figure 6).

## Discussion

Dealing with cancer can be difficult due to the physical and emotional changes it causes affecting the quality of life of patients. Chemotherapy, while effective, can also lead to serious side effects and is expensive. To test potential anti-tumor compounds, we used the ESC tumor model, which involves implanting Ehrlich cells into rats' thigh muscles. Throughout the experiment, we monitored the tumor growth by measuring the volume and weight of the tumor after sacrificing the rats. Our research focused on the potential of echinacoside as a chemotherapeutic agent. Treatment with echinacoside significantly reduced tumor volume and weight and increased the rats' mean survival time from 27 to 48 days. Additionally, we observed improvements in the muscle cell structures under an electron microscope.

We began our investigation by analyzing the PI3K/mTOR pathway, which is known to play a significant role in the survival of breast tumor cells, resistance to apoptosis, and angiogenesis. This pathway can be activated either directly through elevated levels of ROS and VEGF or indirectly through the release of histamine [16]. Autophagy inhibition is a function of mTOR, which enhances the signaling pathways of MAPK and AKT [8]. Inhibiting PI3K and mTOR has shown promising results in combating tumor cells [6]. Our study discovered that treating ESC rats with echinacoside effectively decreased the expression of both PI3K and mTOR. Previous studies have shown that echinacoside has direct inhibitory effects on the PI3K/mTOR pathway, leading to antidepressant effects in mice [17] and ameliorating LPS-induced cell apoptosis and inflammation in rats [18]. However, our study is the first to demonstrate echinacoside's ability to reduce the expression of PI3K/mTOR in ESC rats.

Our study focused on the effects of hypoxia on ESC cells and how echinacoside plays a role in this process. HIF-1α is a key protein marker that is activated during hypoxia and has a short half-life. Once activated, it can contribute to cancer tumorigenicity and progression. During hypoxia, HIF-1α moves into the nucleus and helps the cell adjust its metabolism to the new conditions by linking to DNA [19]. In normal cells, HIF-1α is present in small amounts, but it is highly activated in many tumors [20]. Our research discovered that HIF-1α expression increased significantly in ESC cells but was downregulated after echinacoside treatment. Nonetheless, no prior studies have explored the impact of echinacoside on HIF-1α in ESC cells.

In our study, we investigated the pathway involving cyclin D1/CDK2. Cyclin D1 is responsible for regulating the growth of cells and is often overexpressed in tumors. We observed the expression of cyclin D1 in MC3T3-E1 cells that have undergone osteogenic differentiation [21]. CDK2 is activated by binding with cyclins and plays a crucial role in various biological processes such as signal transduction, DNA damage, intracellular transport, protein degradation, DNA/RNA translation, and metabolism. CDK2 regulates the cell cycle from the late G1-phase and throughout S-phase by interacting with and phosphorylating proteins in cell signaling pathways, which can lead to cancer cell proliferation [22]. Our research revealed that ESC significantly increased the expression of both cyclin D1 and CDK2. However, when treated with echinacoside, these effects were significantly attenuated. Previous studies have reported echinacoside to reduce the expression of cyclin D1 in breast cancer cells [23], but this is the first study to demonstrate its ability to reduce the expression of CDK2.

Finally, we aimed to investigate how echinacoside affects the inflammatory pathway in ESC. Inflammation is usually influenced by a transcription regulator called NFκB, which can be activated by different stimuli like cytokines, free radicals, or viral and bacterial products [24]. TNF-α is a cytokine that plays a vital role in immunity, protection, and inflammation. It is also associated with tumor progression and carcinogenesis [25]. Our findings show that echinacoside significantly reduced the overexpression of both NFκB and TNF-α induced by ESC. Previous studies have reported echinacoside's ability to improve inflammation in different models like depression [26] and Parkinson's disease [27]. However, our study is the first to demonstrate echinacoside's effectiveness in ESC.

Limitations of the study

**Figure 7:**
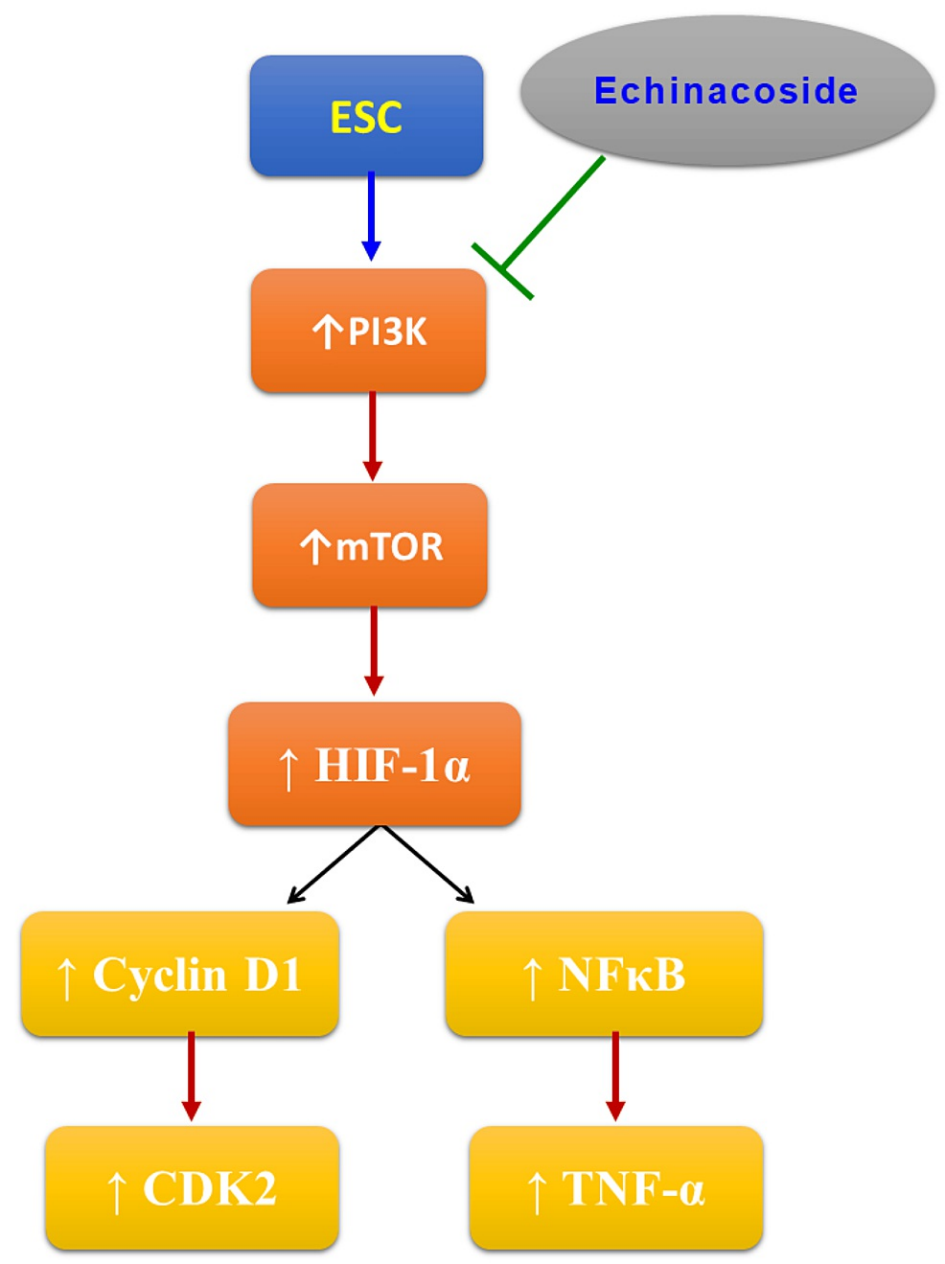
The mechanism of the protective effects of echinacoside in ESC. ESC: Ehrlich solid carcinoma; CDK2: Cyclin-dependent kinase 2; HIF-1α: Hypoxia-inducible factor-1α; mTOR: mammalian target of rapamycin; NFκB: Nuclear factor κB; PI3K: Phosphoinositide-3-kinases; TNF-α: Tumor necrosis factor-α. The image is created by the authors of this study.

Echinacoside is a natural and affordable option for chemotherapy that shows promise as an alternative treatment for ESC. Figure 7 illustrates its therapeutic effects. However, it's important to note that current research has some limitations. For example, rats have different metabolic processes than humans, which can result in varying drug effects. Furthermore, while there are many animal models used for cancer induction, our study only utilized one method.

## Conclusions

Our research confirms that echinacoside possesses potent antitumor properties, resulting in a significant reduction in both tumor size and weight. This leads to a remarkable improvement in muscle structure and a substantial increase in the average survival time of rats. Moreover, echinacoside effectively ameliorates hypoxia by suppressing HIF-1α, reduces inflammation by decreasing NFκB and TNF-α, decreases proliferation by reducing PI3K, and effectively blocks cyclin D1 and CDK2 to inhibit differentiation.
